# Molecular epidemiology and virulence characteristics of *Staphylococcus aureus* nasal colonization in medical laboratory staff: comparison between microbiological and non-microbiological laboratories

**DOI:** 10.1186/s12879-018-3024-x

**Published:** 2018-03-12

**Authors:** Xiaoying Xie, Xinlu Dai, Lijia Ni, Baiji Chen, Zhaofan Luo, Yandan Yao, Xiquan Wu, Hongyu Li, Songyin Huang

**Affiliations:** 1 Department of Clinical Laboratory, Sun Yat-Sen Memorial Hospital, Sun Yat-Sen University, Guangzhou, 510120 China; 2 Department of Clinical Laboratory, The Seventh Affiliated Hospital of Sun Yat-Sen University, Shenzhen, 518071 China; 3 Department of Clinical Laboratory, Zengcheng District People Hospital of Guangzhou, Guangzhou, 511300 China; 4 Department of Laboratory, Guangdong Provincial Key Laboratory of Malignant Tumor Epigenetics and Gene Regulation, Sun Yat-Sen Memorial Hospital, Sun Yat-Sen University, Guangzhou, 510120 China

**Keywords:** *Staphylococcus aureus*, Nasal carriage, Medical laboratory, Antimicrobial susceptibility, Virulence genes, *spa* type

## Abstract

**Background:**

Medical laboratory staff are a high-risk population for colonization of *Staphylococcus aureus (S. aureus)* due to direct and dense contact with the pathogens; however, there is limited information about this colonization. This study sought to determine the prevalence and molecular characteristics of nasal colonization by *S. aureus* in medical laboratory staff in Guangzhou, southern China, and to compare the differences between microbiological laboratory (MLS) and non-microbiological laboratory (NMLS) staff.

**Methods:**

*S. aureus* colonization was assessed by nasal swab cultures from 434 subjects, including 130 MLSs and 304 NMLSs from 33 hospitals in Guangzhou. All *S. aureus* isolates underwent the antimicrobial susceptibility test, virulence gene detection and molecular typing.

**Results:**

The overall prevalence of *S. aureus* carriage was 20.1% (87/434), which was higher in MLSs than in NMLSs (26.2% vs. 17.4%, *P* < 0.05), while the prevalence of Methicillin-resistant *S. aureus* (MRSA) was similar. Living with hospital staff was associated with *S. aureus* carriage. The majority of the isolates harboured various virulence genes, and those in MLSs appeared less resistant to antibiotics and more virulent than their counterparts. A total of 37 different *spa* types were detected; among these, t338, t437, t189 and t701 were the most frequently encountered types. T338 was the main *spa* type contributing to nasal colonization Methicillin-sensitive *S. aureus* (MSSA) (13.0%), and t437-SCC*mec* IV was predominant in MRSA isolates (40%).

**Conclusions:**

These findings provide insight into the risk factors, molecular epidemiology and virulence gene profiles of *S. aureus* nasal carriage among the medical laboratory staff in Guangzhou.

**Electronic supplementary material:**

The online version of this article (10.1186/s12879-018-3024-x) contains supplementary material, which is available to authorized users.

## Backgrounds

*Staphylococcus aureus* (*S. aureus*) is a life-threatening pathogen and a part of the commensal flora, and its antimicrobial resistance has been a significant therapeutic challenge. Methicillin-resistant *Staphylococcus aureus* (MRSA) is of great concern because of its high mortality following treatment failure. *S. aureus* infections are usually preceded by colonization, which is most often found in the anterior nares [1]. Nasal carriage is associated with *S. aureus* (including MRSA) exposure and an increased risk of transmission and infection [2, 3].

*S. aureus* sources and transmission patterns have been extensively investigated, especially in hospital environments [4]. Our previous studies showed possible spread from HA-MRSA (hospital-acquired methicillin-resistant *Staphylococcus aureus*); to CA-MRSA (community-acquired methicillin-resistant *Staphylococcus aureus*), which may make the strains more resistant to antibiotics [5]. Therefore, nasal colonization of HA-MRSA strains in HCWs (healthcare workers) may also play a role in MRSA spread from hospital settings to the community [5]. Several studies about healthcare workers have focused on the clinical staff, such as doctors, nurses and general practitioners [4, 6], while few studies have investigated the medical laboratory staff in the hospital.

Unlike the clinical staff members who have close contact with patients, medical laboratory staff members are exposed to the infectious materials more often, including patients’ blood, fluids and tissues. The medical microbiological laboratory is an important department in the medical lab, and its main function is to isolate, culture and identify the pathogens from the patient specimens. Therefore, the microbiological lab staff members have a higher risk of exposure to *S. aureus* because of their direct and dense contact with pathogens [7]. Therefore, it is important to detect nasal colonization of *S. aureus* among the healthy medical laboratory staff, especially the medical microbiological lab staff.

The objective of this study was to assess the prevalence and factors associated with *S. aureus* and MRSA nasal colonization among medical laboratory staff in hospitals in Guangzhou, China. To the best of our knowledge, this is the first study performed in this population in China. Additionally, some *S. aureus* clones are more virulent than others, although any *S. aureus* genotype can become a pathogen under favourable host conditions. Molecular typing is helpful for supporting infection control measures, investigating suspected outbreaks, and preventing nosocomial transmission [8–11]. Therefore, in this study, all isolates were further genotyped by *spa* typing, and the MRSA isolates were evaluated with SCC*mec* typing. The presence of virulence genes was determined to establish their possible relationship to the epidemiological data, including the enterotoxin, haemolysin, *pvl*, *tsst-*1 and exfoliative genes as well as various adhesive protein producing genes. To date, this is the first study to indicate that medical laboratory staff may have occupational exposure to healthcare-associated *S. aureus* in Guangzhou, China. This knowledge will improve our understanding of the characteristics of *S. aureus* nasal carriage among healthy medical laboratory staff and provide a possible mode of *S. aureus* transmission in our region.

## Methods

### Population and study design

**Fig. 1:**
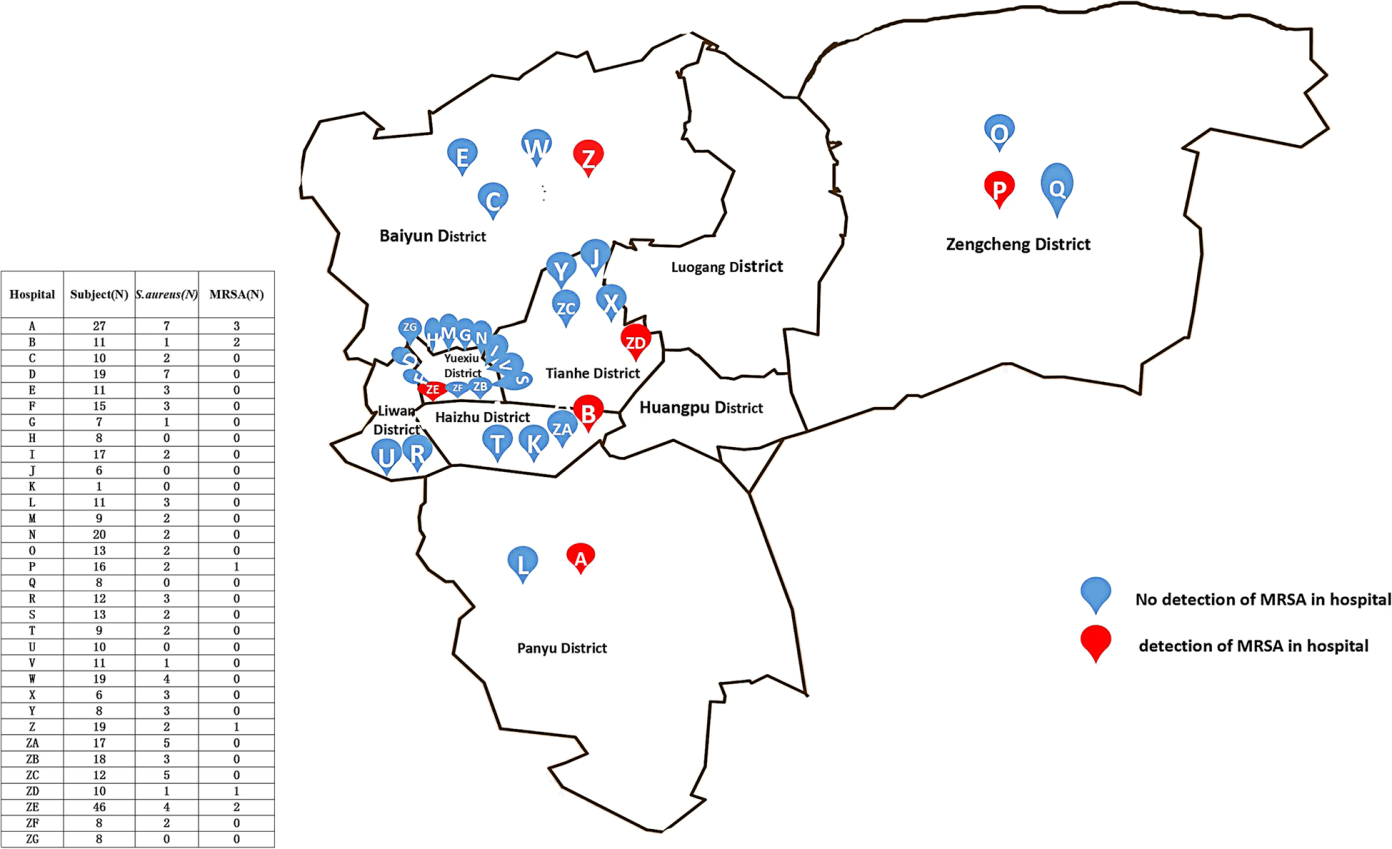
Map of the 33 hospitals in Guangzhou, China, and the subjects, *S. aureus* and MRSA isolates distribution

A cross-sectional prevalence study was conducted in 33 hospitals’ medical laboratories in Guangzhou, China between February 22, 2016, and March 17, 2016. All the subjects were the staff working in medical laboratory; the ones with acute and chronic respiratory tract infection were excluded. As the main metropolises with a large population of more than 10,000,000 in southern China, Guangzhou holds a prominent position in the healthcare of local and surrounding areas. As the main tertiary hospitals, the 33 hospitals are distributed in urban and suburban in Guangzhou, making them representative (Fig. 1).

A total of 434 medical laboratory staff, aged 20–56 years, in the 33 hospitals participated in this study; 130 microbiological laboratory staffs (MLS) were randomly recruited from the microbiological labs, and 304 non-microbiological laboratory staffs (NMLS) were randomly recruited from the non-microbiological labs. All volunteers signed informed consent documents approving the use of their samples for research purposes, and the Ethics Committee of Sun Yat-Sen Memorial Hospital approved the study.

Using a standardized questionnaire, the pertinent demographic, medical information and potential factors that are related to *S. aureus* nasal colonization and transmission were collected from each participant. The “Standard working protection” meant the basic lab rule of hygiene, including hand washing, use of gloves, long sleeves and masks. Before sampling, the subjects were independently required to finish the questionnaires. No surveys were eliminated during data collation, and 434 nasal swabs were sampled.

### Bacterial strains

Both anterior nares were swabbed by rotating a sterile dry cotton swab 5 times inside the nostril. The samples were immediately stored in Copan eSwab Liquid Amies preservation medium (eSwab Collection and Preservation System, Copan Italia, Brescia, Italy) and transported at room temperature to the department of bacteriology in 4 h. The swabs were streaked on blood agar plates at 35 °C for 24 h. Gram-positive, β-haemolytic, coagulase-positive isolates were confirmed as *S. aureus* using a Vitek®2 microbial identification system (bioMérieux, Marcy l’Etoile, France) according to the manufacturer’s instructions.

### Antimicrobial susceptibility testing

Antimicrobials used for susceptibility testing included penicillin, erythromycin, clindamycin, tetracycline, cefoxitin, chloramphenicol, rifampicin, ciprofloxacin, gentamicin, trimethoprim/sulfamethoxazole, teicoplanin, linezolid, levofloxacin and vancomycin. Susceptibilities were determined using the disk diffusion method in accordance with the performance standards for antimicrobial susceptibility testing, 26th informational supplement (M100-S26), recommended by the Clinical and Laboratory Standards Institute. The susceptibility for vancomycin was determined using the E-test method. Inducible clindamycin resistance was determined by the D-test. All disks and E-tests were obtained from Oxoid Ltd. (Oxoid, Basingstoke, England), and *S. aureus ATCC 25923* and *ATCC29213* were used as the quality control strains.

### Molecular characterization

Bacterial DNA was extracted using a DNA extraction kit (Tiangen Biotech, Beijing) with lysostaphin according to the manufacturer’s instructions. PCR of the *spa* locus with subsequent sequencing was performed on the 87 *S. aureus* isolates, including clustering of the *spa* types into *spa* clonal complexes (using the Ridom Staph Type version 1.5 software package, www.spaserver.ridom.de) as previously reported [12]. SCC*mec* typing of MRSA strains was conducted by a multiplex PCR method as previously described [13]. The presence of the genes that code for Panton-Valentine leukocidin (*lukF-PV/lukS-PV* or *pvl*), twelve staphylococcal enterotoxins (*sea~see, seg~sej* and *sem-seo*), two exfoliative toxins (*eta* and *etb*), four haemolysins (*α-hemolysin, β-hemolysin, δ-hemolysin,* and *γ-hemolysin*), three biofilm information-related genes (*ica A, ica D* and *bap*), and the toxic shock syndrome toxin (*tsst-*1) and *mec*A gene were determined using PCR as previously reported, with some modification [14, 15].

### Statistical analysis

In descriptive statistics, the frequency and proportions were calculated for categorical variables. The frequency of the SCC*mec* type, specimen type and virulence genes were treated as categorical variables. The chi-square or two-sided Fisher’s exact test was used to discriminate whether the distributions of the studied genes or types were significantly different between the MLS and NMLS groups. The only continuous variable, the duration of employment, was transformed into a categorical variable using the quartiles of the frequency distribution (< 1, 1~ 5, 6~ 10, 11~ 20, and > 20). The odds ratios (OR), 95% confidence intervals (CI), and *P*-values were calculated. A finding was considered statistically significant for a two-sided *P*-value< 0.05. Univariable logistic regression models were applied to determine the independent risk factors. Multiple logistic regression analysis was performed by stepwise backward selection of variables with biological plausibility and a significance level < 0.10 for entry into the model. All statistical analyses were performed using SPSS 19.0 for Windows (IBM).

## Results

### *S. aureus* Nasal colonization

A total of 434 medical laboratory staff members in the 33 hospitals were enrolled in this study. The demographic characteristics of the study population are shown in Table 1. The average age of the participating volunteers was 34.2 ± 12.2 years, and 187 (43.1%) of the volunteers were male. *S. aureus* was detected in the nasal swabs of 87 participants (20.0%, 87/434). The overall prevalence of *S. aureus* nasal colonization was 26.2% (34/130) in MLS, which was higher than that in NMLS (17.4%, 53/304) (*P* = 0.03) (OR = 1.677, 95% CI: 1.027–2.740). Additionally, there were no differences for other demographic characteristics and potential risk factors between the two comparison groups. A proper nasal cavity cleaning habit could help to decrease the *S. aureus* nasal carriage [16], but it is interesting to note that the *S. aureus* nasal carriage rate in people who cleaned their nasal cavity daily or weekly was higher than for those who rarely or never cleaned their nasal cavity (*P* = 0.032) (OR = 1.753, 95% CI: 1.050–2.927) in this study, and those living with hospital staff were more likely to be colonized by *S. aureus* in the nasal cavity (*P* = 0.008) (OR = 1.909, 95% CI: 1.182–3.083). In multiple logistic regression analysis, nasal colonization of *S. aureus* was also significantly associated with working in a microbiological laboratory and living with hospital staff.

**Table 1:** Prevalence of *S. aureus* and MRSA carriage by population group and factors associated with nasal carriage

Characteristic (n)	*S. aureus*	Univariate logistic		Multivariate logistic		MRSA	Univariate logistic	
	n (%)	*P* value	OR(95% CI)	*P* value	OR (95% CI)	n (%)	*P* value	OR(95% CI)
Sex								
Male (187)	42(22.5)	0.155	1.414(0.877–2.279)			4(2.1)	0.842	0.878(0.244–3.157)
Female (247)	42(17.0)					6(2.4)		
Duration of employment								
< 1 year (49)	9(18.4)	0.149	1.790(0.813–3.941)			3(6.1)	0.075	3.522(0.880–14.093)
1–5 years (150)	35(23.3)	0.215	1.358(0.837–2.202)			2(1.3)	0.338	0.466(0.098–2.224)
6–10 years (93)	17(18.2)	0.631	0.866(0.481–1.559)			2(2.2)	0.911	0.915(0.191–4.383)
11–20 years (85)	18(21.2)	0.772	1.090(0.608–1.953)			3(3.5)	0.407	1.787(0.452–7.061)
> 20 years (57)	8(14.3)	0.228	0.616(0.280–1.354)			0(0)	–	–
Department								
Microbiological laboratory (130)	34(26.2)	0.039	1.677(1.027–2.740)	0.04	1.630(1.024–2.597)	2(1.5)	0.492	0.578(0.121–2.760)
Other laboratory (304)	53(17.4)					8(2.6)		
Work protection								
Standard (354)	74(20.9)	0.155	1.620(0.833–3.151)			8(2..3)	0.951	0.951(0.197–4.597)
Non-standard (80)	13(16.2)					2(2.5)		
Nasal cleaning habit								
Daily or weekly (106)	29(27.4)	0.032	1.753(1.050–2.927)	0.101	1.511(0.923–2.471)	3(2.8)	0.679	1.336(0.339–5.259)
Rarely or never (328)	58(17.7)					7(2.1)		
Living with hospital staff								
Yes (147)	40(27.2)	0.008	1.909(1.182–3.083)	0.008	1.848(1.174–2.901)	3(2.0)	0.794	0.833(0.212–3.271)
No (287)	47(16.4)					7(2.4)		
Underlying disease								
Yes (65)	17(26.2)	0.239	1.873(0.982–3.573)			2(3.3)	0.65	1.884(0.386–9.195)
No (369)	70(18.9)					8(2.2)		
Hospitalization in past one years								
Yes (32)	9(28.1)	0.157	1.085(0.450–2.617)			0(0)	–	
No (402)	78(19.4)					10(2.5)		
Current smoking statue								
Yes (23)	5(21.7)	0.837	1.114(0.402–3.090)			1(4.3)	0.511	2.030(0.246–16.748)
No (411)	82(20.0)					9(2.2)		

MRSA, methicillin resistant *S. aureus*

Underlying disease: hypertension, diabetes, chronic rhinitis, urticaria, hyperthyroidism; *OR* odds ratio, *CI* confidence interval

Ten MRSA strains were isolated in this study, two in the MLS group (1.5%, 2/130) and eight in its counterparts (2.6%, 8/304), while no significant differences were found between the two groups (*P* = 0.487). MRSA accounted for 11.5% of all *S. aureus* isolates, and its distribution was centred in three hospitals, Hospital A (three isolates, typed as t008, t437 and t701 respectively), Hospital B (two isolates, typed as t571 and t441 respectively) and Hospital ZE (two isolates, typed as t437 and t037 respectively), with relative hospital clustering. The distributions of *S. aureus* and MRSA carriers stratified by population characteristics are shown in Table 1 and Fig. 1.

### *Spa* typing and SCC*mec* typing

**Fig. 2:**
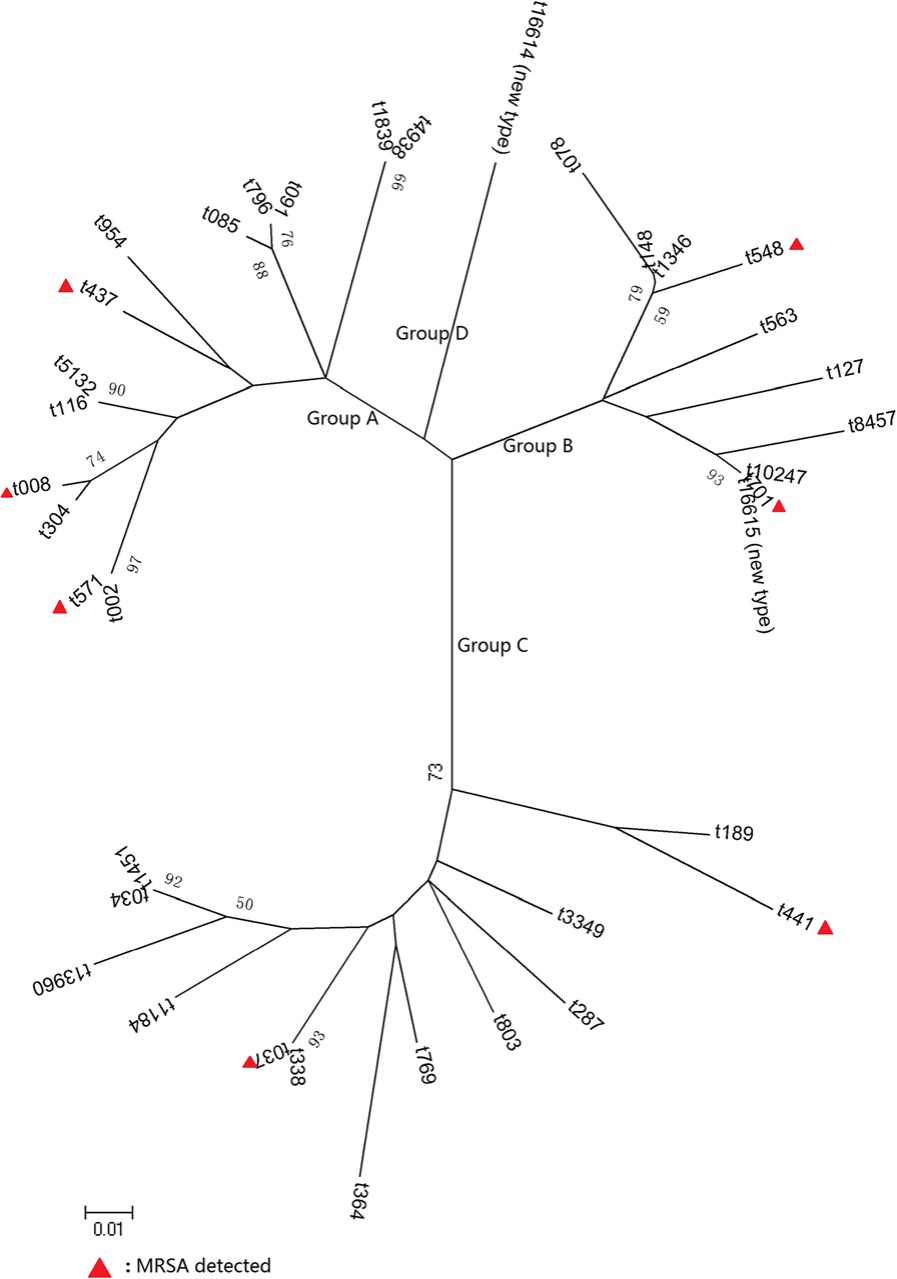
Thirty-seven *spa* types grouped into four groups based on DNA microarray analysis. Group A: t002, t571,t304, t008, t116, t5132, t437, t954, t085, t796, t091, t1839, t4938; Group B: t078, t148, t1346, t548, t563, t127, t8457, t10247, t701, t16615(new type); Group C: t189, t441, t3349, t287, t803, t769; t364, t338, t037, t1184, t13960, t034, t1451; Group D: t16614 (new type)

The 87 *S. aureus* strains were *spa* typed, resulting in 37 different types, including two new types (Fig. 2 and Additional file 1: Table S1). The largest observed *spa* clusters were types t338 (*n* = 10, 11.5%) and t437 (*n* = 10, 11.5%), which were followed by t189 (*n* = 8, 9.2%) and t701 (*n* = 8, 9.2%). All types t338 and t189 were MSSA isolates, and type 701 was also mainly found in the MSSA group. MRSA isolates clustered in *spa* type t437 (4/10, 40%). All other *S. aureus* isolates had a distinct *spa* type. Additionally, two new *spa* types, t16614 (26–23–12-34-12-23-02-02-12-23) (Kreiswirth IDs: TJGBGJAAGJ) and t16615 (11–10–21-17-17-34-24-34-22-25-25) (Kreiswirth IDs: YC2FMMBQBLOO), were found in the MSSA isolates, and they were obtained from nasal swabs of a 26-year-old female non-microbiological lab staff member and a 33-year-old male microbiological lab staff member, respectively. There was no clustering of the isolate *spa* types within different departments.

Based on homology analysis, 37 *spa* types were grouped into four groups (Fig. 2). Group A contained 12 *spa* types, including t437, t571 and t002, which evolved based on t002. Group B consisted of ten types, including t701 and t127. Group C contained 13 types, including t189 and t338. The new *spa* type t16614 formed group D alone. By sequence alignment, the homology of the s*pa* types in the same group was more than 90%. Another new type, t16615, has high homology with t701. The isolates in groups A, B and C were obtained from both the NMLS and MLS groups, while the isolates from the microbiological laboratory were mainly from groups A and C, accounting for 85.3% (29/34). Ten MRSA isolates were dispersed in different groups.

The distribution of SCC*mec* types in the ten MRSA strains is shown in Table 2. Among these, SCC*mec* type IV was found in eight isolates (80.0%), MRSA-t437-IV was the most prevalent of all MRSA isolates (40.0%, 4/10) (Fig. 2 and Table 2). Two MRSA isolates in the NMLS group were SCC*mec* I MRSA and SCC*mec* III MRSA, respectively, which were related to hospital-acquired MRSA (HA-MRSA). As a common virulence factor of community-acquired MRSA (CA-MRSA), the *pvl* gene was positive in 50% of isolates in the ten MRSA isolates.

**Table 2:** Distribution of the ten MRSA strains in details

Strain No.	Hospital	Department	Age/Gender	*spa* type	SCC*mec*	Resistance pattern	Virulence pattern
1	A	NMLS	22/Female	t008	IVa	DA	*sea/tsst-*1*/hemolysis α/γ/δ/icaD/pvl*
2	A	NMLS	20/Female	t437	IVa	–	*sea/seb/she/tsst-*1*/hemolysis α/γ/δ/icaD/pvl*
3	A	NMLS	23/Female	t701	IVd	DA/E /TE	*sea/eta/hemolysis α/γ/δ/icaD/pvl*
4	B	NMLS	34/Female	t571	IVa	DA/E/RD	*seh/tsst-*1*/hemolysis α/γ/δ/icaD*
5	B	NMLS	29/Male	t441	I	DA/E /TE	*seb/tsst-*1*/hemolysis α/β/γ/δ/icaD/ pvl*
6	ZE	NMLS	33/Female	t437	IVa	DA/E	*seb/seh/tsst-*1*/hemolysis α/β/γ/δ/icaD*
7	ZE	NMLS	32/Female	t037	III	DA/E/RD	*sea/seh/tsst-*1*/hemolysis α/β/γ/δ/icaD*
8	P	NMLS	53/Male	t437	IVa	DA/E /C	*seb/seh/tsst-*1*/hemolysis α/β/γ/δ/icaD*
9	Z	MLS	32/Female	t437	IVa	DA/E /TE	*seb/seh/tsst-*1*/hemolysis α/β/γ/δ/icaD/pvl*
10	ZD	MLS	50/Male	t548	IVd	DA/E/ C/TE	*sed/seg/seh/sei/sej/sen/seo/sem/tsst-*1*/hemolysis α/γ/δ/icaD*

MLS, microbiological lab staff; NMLS, non-microbiological lab staff; −,not detected; DA,clindamycin; TE,tetracycline; E,erythromycin; RD, rifampin; C,chloramphenicol; *Sea~seo,* gene encoding staphylococcal enterotoxins; *hla~hlg*, gene encoding α-haemolysin~γ- hemolysin; *tsst*-1, gene encoding toxic shock syndrome toxin 1; *pvl, g*ene encoding Panton-Valentine leukocidin; *icaD*, gene relative with the bio-film formation

### Antimicrobial susceptibility

**Fig. 3:**
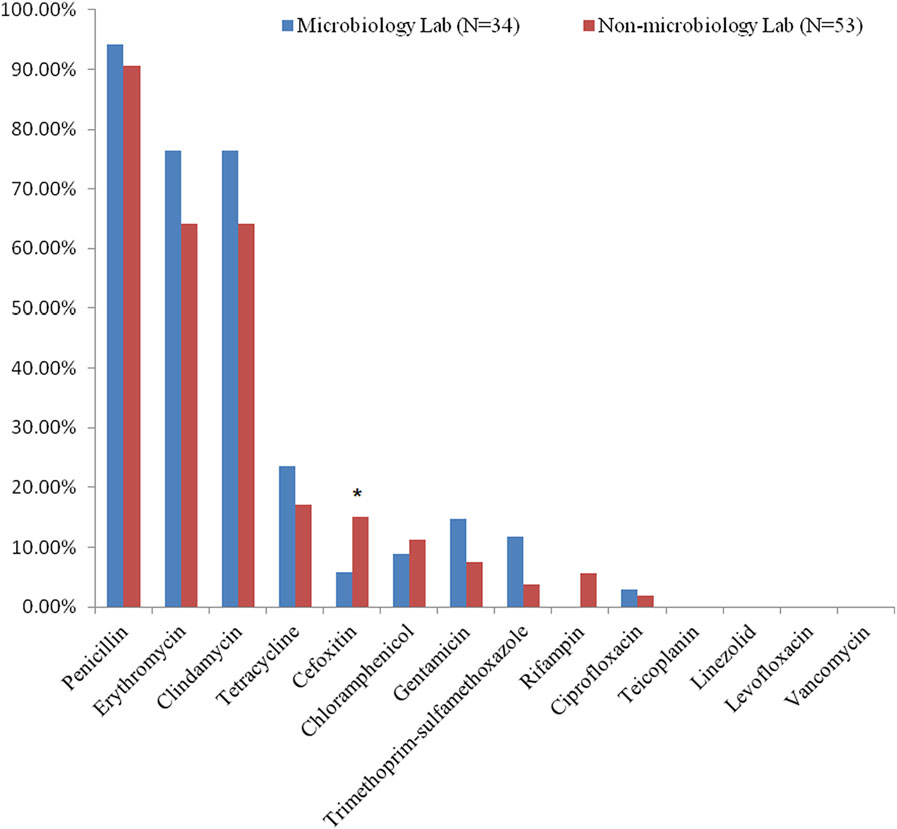
Comparison of the resistance rate to 14 antibiotics of the *S. aureus* isolates between MLS and NMLS group. MLS vs. NMLS, *P < 0.05. MLS, microbiological lab staff; NMLS, non-microbiological lab staff

All isolates were β-lactamases positive. A high resistance rate to penicillin was detected (92.0%, 80/87) in the 87 *S. aureus* isolates, which was followed by the erythromycin (69.0%, 60/87) and clindamycin (69.0%, 60/87). All isolates were susceptible to levofloxacin, vancomycin, teicoplanin and linezolid. A total of 44 (50.6%, 44/87) isolates were MDRSA [17]; 22 in MLS (64.7%, 22/34) and 22 in NMLS (41.5%, 22/53). Penicillin-erythromycin-clindamycin (49.4%, 43/87) was the predominant resistance profile. Only ten isolates (11.5%, 10/87) were resistant to cefoxitin, and they were confirmed to be MRSA by *mecA* PCR screening. Nine MRSA isolates were resistant to erythromycin and clindamycin. Only one MRSA isolate was resistant to erythromycin, clindamycin, tetracycline, and gentamycin. Notably, no MRSA was resistant to quinolone in this study. The resistant percentage to 14 antibiotics of the *S. aureus* isolates is shown in Fig. 3 and Additional file 2: Table S2.

### Virulence factors

Almost all the isolates contained haemolysin gene *hla* (97.7%, 85/87), *hld* (98.9%, 86/87) and *hlg* (98.9%, 86/87) and all isolates in the MLS group were *hla*-*hld*-*hlg* positive. Twelve staphylococcal enterotoxins genes (*sea, seg, seh, sem, sen, seo, sec*, *sei*, *sej*, *sem*, *sen* and *seo*) make up the enterotoxin gene cluster (*egc*). *Sea, seg, seh, sem, sen,* and *seo* genes were found in more than 20% isolates in the 87 *S. aureus* isolates. Additionally, *sec*, *sei* and *sej* genes were mainly found in the 34 *S. aureus* isolates in MLS (17.7%, 23.5% and 11.8% respectively), while they were rarely in NMLS. The *see* gene was not found. Toxic shock syndrome toxin gene (*tsst-*1) was found in 20 isolates (23.0%), and the exfoliative toxins genes (*eta* or *etb*) were rarely found (2.3% and 0, respectively). The *pvl* gene was found in 13 isolates (14.9%), and five isolates were MRSA. The *icaA* and *bap* genes were not found, while another biofilm formation related gene *icaD* widely existed in the *S. aureus* isolates in this study (Table 3).

**Table 3:** Detection of the 23 virulence genes of 87 *S. aureus* isolates in this study

Virulence gene	No. Of positive isolates (*n* = 87) n(%)	No. Distributing in		*P*-value
		Microbiology laboratory (*n* = 34) n(%)	Other laboratory (*n* = 53) n(%)	
*sea*	23(26.4)	8(23.5)	15(28.3)	0.622
*seb*	15(17.2)	4(11.7)	11(20.8)	0.279
*sec*	7(8.1)	6(17.6)	1(1.9)	0.008
*sed*	5(5.8)	2(5.9)	3(5.7)	0.965
*see*	0(0)	0(0)	0(0)	–
*seg*	26(29.9)	12(35.3)	14(26.4)	0.377
*seh*	21(24.2)	9(26.5)	12(22.6)	0.684
*sei*	9(10.4)	8(23.5)	1(1. 9)	0.001
*sej*	4(4.6)	4(11.8)	0(0)	0.011
*sem*	29(33.3)	12(35.3)	17(32.1)	0.756
*sen*	24(27.6)	10(29.4)	14(26.4)	0.76
*seo*	18(20.7)	5(14.7)	13(24.5)	0.27
*eta*	2(2.3)	0(0)	2(3.8)	–
*etb*	0(0)	0(0)	0(0)	–
*hla*	85(97.7)	34(100)	51(96.2)	0.252
*hlb*	38(43.7)	12(35.3)	26(49.1)	0.207
*hld*	86(98.9)	34(100)	52(98.1)	–
*hlg*	86(98.9)	34(100)	52(98.1)	–
*tsst-*1	20(23.0)	6(17.7)	14(26.4)	0.343
*pvl*	13(14.9)	7(20.6)	6(11.3)	0.237
*icaA*	0(0)	0(0)	0(0)	–
*icaD*	77(88.5)	31(91.2)	46(86.8)	0.734
*bap*	0(0)	0(0)	0(0)	–

*Sea~seo,* gene encoding staphylococcal enterotoxins; *eta* and *etb*, gene encoding exfoliatin; *hla~hlg*, gene encodingα-hemolysin~γ-hemolysin; *tsst*-1, gene encoding toxic shock syndrome toxin 1; *pvl*, *g*ene encoding Panton-Valentine leukocidin; *icaA, ica D* and *bap,* gene relative with the bio-film formation

Among isolates in MLS, the detection rates of *sec* and *sei* were higher than in the NMLS group (17.6% vs. 1.9% and 23.5% vs. 1.9%, respectively) (*P* < 0.05), and all *sej* positive isolates were found in the MLS group (11.8%, 4/34). On the other hand, the *eta* gene was only found in isolates of NMLS (3.8%, 2/53). Furthermore, more than half (51.7%) of the 87 *S. aureus* isolates carried more than five virulence genes, and 26.5% (9/34) of the 34 isolates in MLS group carried more than seven virulence genes, which were obviously higher than that in the NMLS group (18.9%, 10/53) (*P* < 0.05). Additionally, 80% of MRSA (8/10) carried more than five virulence genes, which was obviously higher than in MSSA (48.05%, 37/77) (*P* < 0.05) (Table 3).

## Discussion

To the best of our knowledge, this is the first study that focused on the prevalence of *S. aureus* and MRSA colonization in medical laboratory staff in China, especially a comparison between microbiological laboratory and non-microbiological laboratory staff. This population had a high risk of *S. aureus* colonization. The overall prevalence of *S. aureus* colonization was 20.0% in this study. Compared with the general population, this is higher than the recorded prevalence among healthy adults in northern China (16.5%) and in adults in community settings in southern China (13.7%) [18, 19], while it agrees with the rates in America and Europe (20–30%) [20]. Compared with the other HCWs (working mainly in the hospital), the prevalence of *S. aureus* colonization in this study coincides with a review that calculated 23.7% of 10,589 HCWs worldwide carried *S. aureus* [4]. We expected a higher prevalence of *S. aureus* colonization in the MLS, as there is a higher risk of exposure to *S. aureus* because of the direct, dense contact with pathogens. The MLSs in this study had a high prevalence of *S. aureus* colonization (26.2%), which was significantly higher than the NMLSs (non-microbiological laboratory staff) (17.4%) and higher than that in our other study that focused on the prevalence of *S. aureus* colonization in the nurses and clinical doctors in Guangzhou (21.6%) [16]. The MRSA colonization rate in this study (2.3%) was higher than that in northern Europe (0.2%) and southern China (0.3%) for healthy adults [21], and it was higher than the 1.0% in our other study on clinical nurses and doctors [16]. But both the colonization rate of *S. aureus* and MRSA were lower than the research about the carriage among health care professionals (HCPs) attending an international symposium (32.4% and 5.3%, respectively), 66.88% of which subjects worked in MLS, and major of which came from Western Europe [7].

Youth, male gender and chronic disease were the most significant risk factors for nasal *S. aureus* colonization in the healthy population according to past studies [2, 18]; however, in our study, there were no significant differences between different ages and underlying disease conditions. For the medical lab staff, our study found that higher *S. aureus* colonization rates were associated with living with hospital staff. Hospital staff had high risks of pathogen carriage and could act as a “vector” and transmit *S. aureus* to their family, roommates and colleagues [4]. In this study, the prevalence of *S. aureus* colonization appeared to have relative area and hospital clustering, especially MRSA, 70% of which come from three hospitals, and two isolates were detected in two student technicians in training at Hospital A who were roommates.

The *spa* types revealed a broad range of types with a few more prevalent ones, such as t338, t437, t189 and t701, in this study. There were some unique *spa* clusters in MSSA (t338, t189 and t701) and MRSA (t437) isolates. As the main *spa* clusters of MSSA in this study, t189 and t338 had high homology, which coincides with the past reports about the molecular epidemiology in seven main cities in China during this time [22]. As a variant of t002, t437 was the predominant *spa* type of MRSA. According to past reports, MLST CC59-MRSA, which is primarily linked with the *spa* type t437, was the predominant community-associated MRSA clone in Asia, and it represented a genetically tight cluster in Europe [23]. The main SCC*mec* type of MRSA in this study was SCC*mec* IV, including IVa and IVd, which showed the isolates were mainly CA-MRSA. Combined with *spa* typing, MRSA-t437-IV was the most prevalent of all MRSA isolates, which was reported to be a prevalent clone in the community setting in northern China and Taiwan [24, 25]. Two new *spa* types, t16614 and t16615, were detected in this study; both isolates were detected in MSSA isolates and had characteristics of low virulence and low resistance to antibiotics. Although the *spa* types are evolving over time in the same person [1, 26, 27], the clustering of the *spa* type in *spa* complexes could also be used to describe the transmission direction in our study because all sampling was performed within a short time period (three weeks). There was no obvious difference in the molecular type between MLS and NMLS.

According to the antimicrobial susceptibility test, we found there was a high rate of resistance to penicillin, erythromycin and clindamycin and more than half of the isolates had MDR (multi drug resistance). This resistance pattern is associated with the excessive use of penicillin and macrolides in China and requires substantial attention [28, 29]. *Spa* types t189 and t701 appeared relatively sensitive to antibiotics, and their main resistance pattern was to penicillin. While t338 and t437 isolates had higher resistance, and their main resistance pattern was to penicillin-clindamycin-erythromycin.

In contrast to the low prevalence of the exfoliative toxins genes (*eta* or *etb*) and *pvl* gene, the enterotoxin genes, *tsst-*1 gene and haemolysin genes were widely distributed among the isolates; more than a half of the isolates carried over five virulence genes. Previous studies have confirmed that the *sea* and *seb* genes are the most abundant toxin genes in clinical *S. aureus* isolates from patients in China [5, 30, 31]; in this study, *seg* and *sem~n* were the predominant enterotoxin genes, which may be related to the different studied populations. Haemolysin of *S. aureus* contributes to bacterio-lyticenzyme release that can damage the nearby organizations through affecting the red blood cells, platelets and neutrophils [32]. Consistent with a report in China [32], almost all isolates contained *hla*, *hld* and *hlg* genes. The *tsst*-1 gene was found in 23.0% of isolates in this study, suggesting that once infection or cross-infection occurred, these patients may have a greater potential for developing fatal toxic shock syndrome (TSS) [33]. Of note, the isolates in MLS appeared to carry more virulence determinants than the ones in NMLS, which may relate to their more heavy and direct exposure to pathogens. MRSA isolates harboured more virulence genes than MSSA isolates in this study. *Spa* type t189 and t701 isolates harboured fewer virulence genes in this study, and the main toxin pattern was *hemolysis α/γ/δ/icaD* (data not shown)*.* As the predominant *spa* type, t437 appeared more virulent, and it mainly harboured *seb/seh/tsst-1/pvl/hemolysis α/β/γ/δ/icaD* genes. Among the main *spa* types in this study, the most virulent type was t338, which harboured more toxin genes (*sea/seg/ sem~o/tsst-1/hemolysis α/γ/δ/icaD*). Notably, one MRSA isolate typed as t548-SCC*mec* IVd harboured up to 13 virulence genes and appeared to have high resistance to antibiotics; it was isolated from a 50-year-old male microbiology lab staff member who came from hospital ZD, had worked in this department for 27 years and smoked, poor work protection (sometimes didn’t wear mask), rarely nasal cleaning and no underlying diseases; additionally, there were HCWs in his family. This colonization of a healthy medical staff member by a high toxin producing and highly resistant strain is worrisome. In the absence of effective function control practices, these strains may become widely disseminated in the hospital and community.

The current study had several limitations. Most importantly, the small size limited the broad representative significance of the research. Second, sampling of only the nostrils without including other body parts may underestimate the frequency of MRSA colonization. Third, MLST typing was not performed in the current study, and it should be conducted in future analyses. Despite the above limitations, our results confirmed the nasal *S. aureus* colonization in a special population, medical lab staff in local region.

## Conclusions

These findings demonstrate an average prevalence of *S. aureus* nasal colonization in medical lab staff as for other HCWs according to the previous reports. Living with hospital staff members and improper nasal hygiene could increase the colonization risk. Some unique *spa* clusters (t338, t437, t189 and t701), high antibiotic resistance and high virulence were found in the *S. aureus* isolates. Furthermore, MLS had a higher prevalence of *S. aureus* colonization, and the isolates appeared more virulent than the ones in NMLS, which may relate to their more heavy, direct exposure to the pathogens. Some of the many infection prevention partitions, which influence the *S. aureus*/MRSA epidemiology, should be handled by the medical lab staff, especially in the microbiology department, because they have appropriate work protection, nasal clean and hand washing habits.

## Additional files

Additional file 1: **Table S1.** *Spa* types and genetic characteristics of select *S. aureus* isolates recovered from medical laboratory staff. (DOCX 26 kb)

Additional file 2: **Table S2.** The resistance rate to 14 antibiotics of 87 *S. aureus* isolates. (DOCX 20 kb)

## Abbreviations

CA-MRSA: community-acquired methicillin-resistant *Staphylococcus aureus*
CLSI: Clinical and Laboratory Standards Institute
ET: exfoliative toxin
HA-MRSA: hospital-acquired methicillin-resistant *Staphylococcus aureus*
*hla~hlg*: α-hemolysin ~ γ-hemolysin gene
MDRSA: multi-drug resistance *S. aureus*
MLS: microbiological laboratory
MRSA: Methicillin-resistant *S. aureus*
MSSA: Methicillin-sensitive *S. aureus*
NMLS: non-microbiological laboratory staff
PVL: Panton-Valentine leukocidin gene
*S. aureus*: *Staphylococcus aureus*
SCC*mec*: staphylococcal cassette chromosome mec
SE: staphylococcal enterotoxins
*sea~seo*: staphylococcal enterotoxins A~O gene
ST: sequence type
*tsst*-1: toxic shock syndrome toxin 1 gene

## References

[1] Kluytmans J, van Belkum A, Verbrugh H (1997). Nasal carriage of *Staphylococcus aureus*: epidemiology, underlying mechanisms, and associated risks. Clin Microbiol Rev.

[2] Botelho-Nevers E, Berthelot P, Verhoeven PO, Grattard F, Cazorla C, Farizon F, Pozzetto B, Lucht F (2014). Are the risk factors associated with *Staphylococcus aureus* nasal carriage in patients the same than in healthy volunteers? Data from a cohort of patients scheduled for orthopedic material implantation. Am J Infect Control.

[3] Abou Shady HM, Bakr AE, Hashad ME, Alzohairy MA (2015). *Staphylococcus aureus* nasal carriage among outpatients attending primary health care centers: a comparative study of two cities in Saudi Arabia and Egypt. Braz J Infect Dis.

[4] Albrich WC, Harbarth S (2008). Health-care workers: source, vector, or victim of MRSA?. Lancet Infect Dis.

[5] Xie X, Bao Y, Ouyang N, Dai X, Pan K, Chen B, Deng Y, Wu X, Xu F, Li H (2016). Molecular epidemiology and characteristic of virulence gene of community-acquired and hospital-acquired methicillin-resistant *Staphylococcus aureus* isolates in sun Yat-sen memorial hospital, Guangzhou, southern China. BMC Infect Dis.

[6] Hoffmann K, den Heijer CD, George A, Apfalter P, Maier M (2015). Prevalence and resistance patterns of commensal S. Aureus in community-dwelling GP patients and socio-demographic associations. A cross-sectional study in the framework of the APRES-project in Austria. BMC Infect Dis.

[7] Saadatian-Elahi M, Tristan A, Laurent F, Rasigade JP, Bouchiat C, Ranc AG, Lina G, Dauwalder O, Etienne J, Bes M (2013). Basic rules of hygiene protect health care and lab workers from nasal colonization by *Staphylococcus aureus*: an international cross-sectional study. PLoS One.

[8] Enright MC, Robinson DA, Randle G, Feil EJ, Grundmann H, Spratt BG (2002). The evolutionary history of methicillin-resistant *Staphylococcus aureus* (MRSA). Proc Natl Acad Sci U S A.

[9] Liu Y, Wang H, Du N, Shen E, Chen H, Niu J, Ye H, Chen M (2009). Molecular evidence for spread of two major methicillin-resistant *Staphylococcus aureus* clones with a unique geographic distribution in Chinese hospitals. Antimicrob Agents Chemother.

[10] Nemeghaire S, Argudin MA, Haesebrouck F, Butaye P (2014). Epidemiology and molecular characterization of methicillin-resistant *Staphylococcus aureus* nasal carriage isolates from bovines. BMC Vet Res.

[11] Mirzaii M, Emaneini M, Jabalameli F, Halimi S, Taherikalani M (2015). Molecular investigation of *Staphylococcus aureus* isolated from the patients, personnel, air and environment of an ICU in a hospital in Tehran. J Infect Public Health.

[12] den Heijer CD, van Bijnen EM, Paget WJ, Pringle M, Goossens H, Bruggeman CA, Schellevis FG, Stobberingh EE, Team AS (2013). Prevalence and resistance of commensal *Staphylococcus aureus*, including meticillin-resistant S aureus, in nine European countries: a cross-sectional study. Lancet Infect Dis.

[13] Chen X, Yang HH, Huangfu YC, Wang WK, Liu Y, Ni YX, Han LZ (2012). Molecular epidemiologic analysis of *Staphylococcus aureus* isolated from four burn centers. Burns.

[14] Sudagidan M, Aydin A (2010). Virulence properties of methicillin-susceptible *Staphylococcus aureus* food isolates encoding Panton-valentine Leukocidin gene. Int J Food Microbiol.

[15] Casagrande Proietti P, Coppola G, Bietta A, Luisa Marenzoni M, Hyatt DR, Coletti M, Passamonti F (2010). Characterization of genes encoding virulence determinants and toxins in *Staphylococcus aureus* from bovine milk in Central Italy. J Vet Med Sci.

[16] Chen B, Dai X, He B, Pan K, Li H, Liu X, Bao Y, Lao W, Wu X, Yao Y, et al. Differences in *Staphylococcus aureus* nasal carriage and molecular characteristics among community residents and healthcare workers at sun Yat-Sen university, Guangzhou, southern China. BMC Infect Dis. 15:303. 10.1186/s12879-015-1032-7 PMC4520063 26223250

[17] Ge B, Mukherjee S, Hsu CH, Davis JA, Tran TTT, Yang Q, Abbott JW, Ayers SL, Young SR, Crarey ET (2017). MRSA and multidrug-resistant *Staphylococcus aureus* in U.S. retail meats, 2010-2011. Food Microbiol.

[18] Yan X, Song Y, Yu X, Tao X, Yan J, Luo F, Zhang H, Zhang J, Li Q, He L (2015). Factors associated with *Staphylococcus aureus* nasal carriage among healthy people in Northern China. Clin Microbiol Infect.

[19] Wang JT, Liao CH, Fang CT, Chie WC, Lai MS, Lauderdale TL, Lee WS, Huang JH, Chang SC (2009). Prevalence of and risk factors for colonization by methicillin-resistant *Staphylococcus aureus* among adults in community settings in Taiwan. J Clin Microbiol.

[20] Mainous AG, Hueston WJ, Everett CJ, Diaz VA (2006). Nasal carriage of Staphylococcus aureus and methicillin-resistant *S aureus* in the United States, 2001-2002. Ann Fam Med.

[21] Walsh EE, Greene L, Kirshner R (2011). Sustained reduction in methicillin-resistant *Staphylococcus aureus* wound infections after cardiothoracic surgery. Arch Intern Med.

[22] Chen Y, Liu Z, Duo L, Xiong J, Gong Y, Yang J, Wang Z, Wu X, Lu Z, Meng X (2014). Characterization of *Staphylococcus aureus* from distinct geographic locations in China: an increasing prevalence of spa-t030 and SCCmec type III. PLoS One.

[23] Glasner C, Pluister G, Westh H, Arends JP, Empel J, Giles E, Laurent F, Layer F, Marstein L, Matussek A (2015). *Staphylococcus aureus* spa type t437: identification of the most dominant community-associated clone from Asia across Europe. Clin Microbiol Infect.

[24] Ho CM, Lin CY, Ho MW, Lin HC, Chen CJ, Lin LC, Lu JJ (2016). Methicillin-resistant *Staphylococcus aureus* isolates with SCCmec type V and spa types t437 or t1081 associated to discordant susceptibility results between oxacillin and cefoxitin, Central Taiwan. Diagn Microbiol Infect Dis.

[25] Chen X, Sun K, Dong D, Luo Q, Peng Y, Chen F (2016). Antimicrobial resistance and molecular characteristics of nasal *Staphylococcus aureus* isolates from newly admitted inpatients. Annals of laboratory medicine.

[26] Golubchik T, Batty EM, Miller RR, Farr H, Young BC, Larner-Svensson H, Fung R, Godwin H, Knox K, Votintseva A (2013). Within-host evolution of *Staphylococcus aureus* during asymptomatic carriage. PLoS One.

[27] Halablab MA, Hijazi SM, Fawzi MA, Araj GF (2010). *Staphylococcus aureus* nasal carriage rate and associated risk factors in individuals in the community. Epidemiol Infect.

[28] Mehndiratta PL, Gur R, Saini S, Bhalla P (2010). *Staphylococcus aureus* phage types and their correlation to antibiotic resistance. Indian J Pathol Microbiol.

[29] Treesirichod A, Hantagool S, Prommalikit O (2014). Nasal carriage and antimicrobial susceptibility of *Staphylococcus aureus* among medical students at the HRH Princess Maha Chakri Sirindhorn medical center, Thailand: a follow-up study. J Infect Public Health.

[30] He W, Chen H, Zhao C, Zhang F, Li H, Wang Q, Wang X, Wang H (2013). Population structure and characterisation of *Staphylococcus aureus* from bacteraemia at multiple hospitals in China: association between antimicrobial resistance, toxin genes and genotypes. Int J Antimicrob Agents.

[31] Wu D, Li X, Yang Y, Zheng Y, Wang C, Deng L, Liu L, Li C, Shang Y, Zhao C (2011). Superantigen gene profiles and presence of exfoliative toxin genes in community-acquired meticillin-resistant *Staphylococcus aureus* isolated from Chinese children. J Med Microbiol.

[32] Liu Q, Han L, Li B, Sun J, Ni Y (2012). Virulence characteristic and MLST-agr genetic background of high-level mupirocin-resistant, MRSA isolates from shanghai and Wenzhou, China. PLoS One.

[33] Chen X, Wang WK, Han LZ, Liu Y, Zhang H, Tang J, Liu QZ, Huangfu YC, Ni YX (2013). Epidemiological and genetic diversity of *Staphylococcus aureus* causing bloodstream infection in shanghai, 2009-2011. PLoS One.

