# Supplementary material for: Molecular epidemiology and virulence characteristics of Staphylococcus aureus nasal colonization in medical laboratory staff: comparison between microbiological and non-microbiological laboratories
Source: BMC Infect Dis. 2018 Mar 12;18:122. doi: 10.1186/s12879-018-3024-x (PMC5848597; doi:10.1186/s12879-018-3024-x)
Supplement: Supplementary file 1 — Table S1. Spa types and genetic characteristics of select S. aureus isolates recovered from medical laboratory staff. (DOCX 26 kb) [file 12879_2018_3024_MOESM1_ESM.docx]

Table S1. *Spa* types and genetic characteristics of select *S. aureus* isolates recovered from medical laboratory staffs.

| *Spa* type | Group | No. Of positive isolates  （n=87) n% | No. distributing in department | | | |
| --- | --- | --- | --- | --- | --- | --- |
|  |  |  | Microbiology laboratory  (n=34) n% | Other laboratory (n=53) n% | MSSA  （n=77) n% | MRSA  (n=10) n% |
| t338 | C | 10 (11.5) | 5(14.7) | 5(9.4) | 10(13.0 ) | 0(0.0) |
| t437 | A | 10 (11.5) | 4(11.8) | 6(11.3) | 6(7.8) | 4(40.0) |
| t189 | C | 8 (9.2) | 2(5.9) | 6(11.3) | 8(10.4) | 0(0.0)) |
| t701 | B | 8 (9.2) | 3(8.8) | 5(9.4) | 7(9.1) | 1(10.0) |
| t571 | A | 5 (5.8) | 2(5.9) | 3(5.7) | 4(5.2) | 1(10.0) |
| t304 | A | 4 (4.6) | 2(5.9) | 2(3.8) | 4(5.2) | 0(0.0) |
| t441 | C | 3 (3.5) | 0(0.0) | 3(5.7) | 2(2.6) | 1(10.0) |
| t796 | A | 3 (3.5) | 2(5.9) | 1(1.9) | 3(3.9) | 0(0.0) |
| t954 | A | 3 (3.5) | 1(2.9) | 2(3.8) | 3(3.9) | 0(0.0) |
| t034 | A | 2 (2.3) | 1(2.9) | 1(1.9) | 2(2.6) | 0(0.0) |
| t078 | B | 2 (2.3) | 0(0.0) | 2(3.8) | 2(2.6) | 0(0.0) |
| t085 | A | 2 (2.3) | 0(0.0) | 2(3.8) | 2(2.6) | 0(0.0) |
| t091 | A | 2 (2.3) | 1(2.9) | 1(1.9) | 2(2.6) | 0(0.0) |
| t803 | C | 2 (2.3) | 1(2.9) | 1(1.9) | 2(2.6) | 0(0.0) |
| t002 | A | 1 (1.2) | 0(0.0) | 1(1.9) | 1(1.3) | 0(0.0) |
| t008 | A | 1 (1.2) | 0(0.0) | 1(1.9) | 0(0.0) | 1(10.0) |
| t037 | C | 1 (1.2) | 0(0.0) | 1(1.9) | 0(0.0) | 1(10.0) |
| t10247 | B | 1 (1.2) | 0(0.0) | 1(1.9) | 1(1.3) | 0(0.0) |
| t116 | A | 1 (1.2) | 0(0.0) | 1(1.9) | 1(1.3) | 0(0.0) |
| t1184 | C | 1 (1.2) | 1(2.9) | 0(0.0) | 1(1.3) | 0(0.0) |
| t127 | B | 1 (1.2) | 1(2.9) | 0(0.0) | 1(1.3) | 0(0.0) |
| t1346 | B | 1 (1.2) | 0(0.0) | 1(1.9) | 1(1.3) | 0(0.0) |
| t13960 | C | 1 (1.2) | 1(2.9) | 0(0.0) | 1(1.3) | 0(0.0) |
| t1451 | C | 1 (1.2) | 0(0.0) | 1(1.9) | 1(1.3) | 0(0.0) |
| t148 | B | 1 (1.2) | 0(0.0) | 1(1.9) | 1(1.3) | 0(0.0) |
| t1839 | A | 1 (1.2) | 1(2.9) | 0(0.0) | 1(1.3) | 0(0.0) |
| t287 | C | 1 (1.2) | 0(0.0) | 1(1.9) | 1(1.3) | 0(0.0) |
| t3349 | C | 1 (1.2) | 1(2.9) | 0(0.0) | 1(1.3) | 0(0.0) |
| t364 | C | 1 (1.2) | 0(0.0) | 1(1.9) | 1(1.3) | 0(0.0) |
| t4938 | A | 1 (1.2) | 1(2.9) | 0(0.0) | 1(1.3) | 0(0.0) |
| t5132 | A | 1 (1.2) | 1(2.9) | 0(0.0) | 1(1.3) | 0(0.0) |
| t548 | B | 1 (1.2) | 1(2.9) | 0(0.0) | 0(0.0) | 1(10.0) |
| t563 | B | 1 (1.2) | 0(0.0) | 1(1.9) | 1(1.3) | 0(0.0) |
| t769 | C | 1 (1.2) | 1(2.9) | 0(0.0) | 1(1.3) | 0(0.0) |
| t8457 | B | 1 (1.2) | 0(0.0) | 1(1.9) | 1(1.3) | 0(0.0) |
| t16614 | D | 1 (1.2) | 0(0.0) | 1(1.9) | 1(1.3) | 0(0.0) |
| t16615 | B | 1 (1.2) | 1(2.9) | 0(0.0) | 1(1.3) | 0(0.0) |
| Total |  | 87 (100.0) | 34(100.0) | 53(100.0) | 77(100.0) | 10(100.0) |
