# Supplementary material for: Molecular epidemiology and virulence characteristics of Staphylococcus aureus nasal colonization in medical laboratory staff: comparison between microbiological and non-microbiological laboratories
Source: BMC Infect Dis. 2018 Mar 12;18:122. doi: 10.1186/s12879-018-3024-x (PMC5848597; doi:10.1186/s12879-018-3024-x)
Supplement: Supplementary file 2 — Table S2. The resistance rate to 14 antibiotics of 87 S. aureus isolates. (DOCX 20 kb) [file 12879_2018_3024_MOESM2_ESM.docx]

Table S2 The resistance rate to 14 antibiotics of 87 *S. aureus* isolates.

| Antibiotics | Resistance rate (%) | | | *P* -value |
| --- | --- | --- | --- | --- |
|  | Microbiology laboratory  (n=34) | Other laboratory  (n=53) | Overall  (n=87) |  |
| Penicillin | 94.1 | 90.6 | 92.0 | 0.552 |
| Erythromycin | 76.5 | 64.2 | 69.0 | 0.233 |
| Clindamycin | 76.5 | 64.2 | 69.0 | 0.233 |
| Tetracycline | 23.5 | 17.0 | 19.5 | 0.339 |
| Cefoxitin | 5.9 | 15.1 | 11.5 | 0.003 |
| Chloramphenicol | 8.8 | 11.3 | 10.3 | 0.644 |
| Gentamicin | 14.7 | 7.6 | 10.3 | 0.285 |
| Trimethoprim-sulfamethoxazole | 11.8 | 3.8 | 6.9 | 0.586 |
| Rifampin | 0 | 5.7 | 3.5 | - |
| Ciprofloxacin | 2.9 | 1.9 | 2.3 | 0.749 |
| Teicoplanin | 0 | 0 | 0 | - |
| Linezolid | 0 | 0 | 0 | - |
| Levofloxacin | 0 | 0 | 0 | - |
| Vancomycin | 0 | 0 | 0 | - |
